# Identification of retinoic acid-regulated nuclear matrix-associated protein as a novel regulator of gastric cancer

**DOI:** 10.1038/sj.bjc.6605202

**Published:** 2009-08-11

**Authors:** J Li, E K O Ng, Y P Ng, C Y P Wong, J Yu, H Jin, V Y Y Cheng, M Y Y Go, P K F Cheung, M P A Ebert, J Tong, K F To, F K L Chan, J J Y Sung, N Y Ip, W K Leung

**Affiliations:** 1 Institute of Digestive Disease, Li Ka Shing Institute of Health Sciences, The Chinese University of Hong Kong, Hong Kong, China; 2 Department of Biochemistry, Hong Kong University of Science and Technology, Hong Kong, China; 3 Department of Medicine II, Technical University of Munich, Munich, Germany; 4 Department of Anatomical and Cellular Pathology, State Key Laboratory in Oncology in South China, The Chinese University of Hong Kong, Hong Kong, China

**Keywords:** p53, p21, L2DTL, oncogene, tumourigenesis, DTL/RAMP

## Abstract

Background: Retinoic acid-regulated nuclear matrix-associated protein (RAMP) is a WD40 repeat-containing protein that is involved in various biological functions, but little is known about its role in human cancer. This study aims to delineate the oncogenic role of RAMP in gastric carcinogenesis.

Methods: RAMP expression was examined by real-time quantitative RT-PCR, immunohistochemistry and western blotting. Inhibition of RAMP expression was performed by siRNA-mediated knockdown. The functional effects of RAMP on cell kinetics were measured by cell viability assay, colony formation assay and flow cytometry. Cell lines stably expressing RAMP were established to investigate the oncogenic effects of RAMP *in vitro.*

Results: *Ramp* was readily expressed in all seven gastric cancer cell lines and was significantly increased in human gastric cancer tissues when compared with their adjacent non-cancerous tissues (*P*<0.001). In keeping with this, expression of RAMP protein was higher in gastric cancer tissues compared with their adjacent non-cancerous tissues, whereas moderate protein expression were noted in intestinal metaplasia. Knockdown of RAMP in gastric cancer cells significantly reduced cell proliferation (*P*<0.01) and soft agar colony formation (*P*<0.001), but induced apoptosis and G_2_/M arrest. In additional, knockdown RAMP induced cell apoptosis is dependent on functional accumulation of p53 and p21 and induction of cleaved caspases-9, caspases-3 and PARP. Strikingly, overexpression of RAMP promoted anchorage-independent cell growth in soft agar.

Conclusion: Our findings demonstrate that RAMP plays an oncogenic role in gastric carcinogenesis. Inhibition of RAMP may be a promising approach for gastric cancer therapy.

Retinoic acid-regulated nuclear matrix-associated protein (RAMP), also known as human lethal 2 denticleless (L2DTL), or DNA replication factor 2 (CDT2), was first identified to be downregulated during retinoic acid-induced neuronal differentiation of NT2 cell (Cheung *et al*, 2001). The *ramp* gene is located in 1q32.1-32.2 region, and encodes a protein containing five WD40 repeats (WDR), a double DxR box, one KEN-box signal and PEST sequences (Pfleger and Kirschner, 2000; Cheung *et al*, 2001; Angers *et al*, 2006). It has been reported that *ramp* plays a pivotal role in embryonic development (Kurzik-Dumke *et al*, 1996; Liu *et al*, 2007). Moreover, RAMP is associated with enhanced metastatic potential of hepatocellular carcinoma and growth of breast cancer cells (Pan *et al*, 2006; Ueki *et al*, 2008). However, the functional role and significance of RAMP in tumourigenesis are still largely unknown.

Gastric cancer is the leading cause of cancer death in China and the second most common cause of cancer death worldwide (Parkin *et al*, 1999). According to the National Cancer Institute (NCI), more than 700 000 people die of gastric cancer every year. Gastric carcinogenesis is thought to be a multi-step process that involves multiple genetic and epigenetic events. Although it is believed that the oncogenic alterations are common events, the underlying mechanisms have not yet been clarified, and further studies are necessary to identify new aberrant genes. Therefore, we extend our study to determine the biological role and clinical application of RAMP in gastric cancer.

## Materials and methods

### Cell lines and primary gastric cancer tissues

Seven human gastric cancer cell lines (AGS, MKN45, KATO III, NCI-N87, SNU16, SNU1 and MKN28) and normal rat fibroblast cell line (Rat2) were obtained from either the American Type Culture Collection (Rockville, MD, USA) or RIKEN Cell Bank (Tsukuba, Japan). All human gastric cancer cells were maintained in RPMI 1640 medium and Rat2 cells were maintained in DMEM medium, supplemented with 10% fetal bovine serum, 100 U ml^−1^ of penicillin and 100 *μ*g ml^−1^ of streptomycin in humidified atmosphere of 5% CO_2_ at 37°C. Paired primary gastric cancer and their adjacent non-cancerous tissues were collected from 47 gastric cancer patients for real-time RT-PCR analysis. Biopsy tissues were obtained from 150 gastric cancer patients, 10 patients with intestinal metaplasia and 10 healthy subjects for immunohistochemical studies. All gastric tissues were histologically confirmed. All patients provided written informed consent for the use of their tissues. This project was approved by the Joint CUHK-NTE Clinical Research Ethics Committee, Hong Kong and Ethics Committee at the Charité University Hospital, Germany.

### Real-time quantitative RT-PCR

Total RNA was extracted from cell pellets or gastric tissues using the TRIzol reagent (Invitrogen, Carlsbad, CA, USA) following the manufacturer's instruction. RNA concentrations were determined by spectrophotometry. Briefly, 1 *μ*g of total RNA was reverse transcribed by using M-MLV reverse transcriptase (Promega, Madison, WI, USA). The resultant cDNA was quantified by using ABI PRISM 7500 with Power SYBR Green PCR Master Mix (Applied Biosystems, Foster City, CA, USA). The primer sequences for *ramp* are F: 5′-TGGCTCAAGTGATGAAGCTG and R: 5′-GGAGCACAGTAGGAGGTTGC. The real-time quantitative PCR was performed in a total volume of 25 *μ*l containing 0.24 *μ*M each of primers, 12.5 *μ*l of 2 × Power SYBR Green PCR Master Mix and 50 ng of cDNA template. Expression level of *ramp* was normalised to *β*-actin mRNA (primer sequences are F: 5′-GTCTTCCCCTCCATCGTG and R: 5′-AGGGTGAGGATGCCTCTCTT). ΔΔCt was then calculated by subtracting ΔCt of the control from ΔCt of disease. Fold change of gene was calculated by the equation 2^−ΔΔCt^. All PCR reactions were performed in triplicates to ensure reproducibility. Electrophoresis of the PCR products on 1% agarose gels was performed to validate the specific generation of the expected PCR product.

### Tissue microarray construction, immunocytochemistry and immunohistochemistry

Tissue microarrays (TMAs) were constructed from 150 gastric cancer patients. Sections of 5 *μ*m were taken from each tissue array block and affixed to 3-aminopropyl triethoxysilane (APES; Sigma, St Louis, MO, USA) coated slides and air-dried overnight at 37°C. After dewaxing and rehydrating, endogenous peroxidase was quenched with 0.3% hydrogen peroxide for 20 min. Immunohistochemistry (IHC) on TMAs was performed on the Ventana Nex ES automated stainer (Ventana Corporation, Tucson, AZ, USA) using the avidin–biotin detection method. The polyclonal primary antibody against RAMP was generated as described previously (Cheung *et al*, 2001), which was used at a concentration of 1 : 500. The histochemical score was applied to assess both the intensity of staining and the percentage of positive cells. For the intensity, a score of 0 to 3, corresponding to negative, weak, moderate and strong positivity, was recorded. The percentage of positive cells at each intensity was also estimated. A grading score was obtained by multiplying the intensity of staining by the percentage of positive cancer cells as reported previously (Rimm *et al*, 2001; Callagy *et al*, 2003).

Immunocytochemistry (ICC) was performed on gastric cancer cell lines (5 × 10^4^) seeded per poly-L-lysine-coated coverslip and fixed with 4% paraformaldehyde solution. Immunohistochemistry was performed on paraffin-embedded tissue sections. To improve the permeability of antibodies, tissues were passed through a graded series of xylene, washed by water and incubated with 3% hydrogen peroxide in PBS for 10 min. After blocking with non-immunised goat serum, the tissue sections were incubated for 2 h with the RAMP antibody (1 : 500) and then goat anti-rabbit IgG antibody (1 : 400) for 30 min and avidin–biotin complex (Dako A/S, Glostrup, Denmark) for 30 min. After incubation with 0.02% of diaminobenzidin (DAB), sections were stained with hematoxylin. ICC was also performed on gastric cancer cell lines with rabbit anti-human antibody against p53 (1 : 500; Cell Signaling, Danvers, MA, USA).

### Knockdown of RAMP, p53 and p21 by siRNAs

MKN45 cells (2 × 10^5^) and AGS cells (1 × 10^5^) were plated in six-well plate 24 h before transfection. RAMP siRNA, which contained a mixture of four siRNAs with concentration of 25 nM each (Dharmacon, Chicago, IL, USA) was transfected with Oligofectamine Transfection Reagent (Invitrogen) as directed by the manufacturer’s instruction. The sense sequences of four siRNA oligonucleotides targeting four different regions of *ramp* RNA are as follows: siRAMP1, 5′-ACUCCUACGUUCUCUAUUATT-3′; siRAMP2, 5′-GUAUGGGAUUUACGUAAGATT-3′; siRAMP3, 5′-AGAAGGCUUUGUUCGAUUGTT-3′; siRAMP4, 5′-GCUAAUUGCACAGACGAUATT-3′. Two predesigned siRNAs (Qiagen, Hilden, Germany) were used to knockdown *p53* or *p21*, with sequences as follows: sip53, 5′-AAGGAAAUUUGCGUGUGGAGU-3′; sip21, 5′-AAGACCAUGUGGACCUGUCAC-3′. A non-silencing siRNA oligonucleotide, (sense sequence) 5′-CGUACGCGGAAUACUUCGATT-3′ (Ambion, Foster City, CA, USA), targeting the luciferase RNA was used as control.

### Construction of stably RAMP-expressing stable cell lines

The entire coding sequence of RAMP cDNA was cloned into the pcDNA3.1/V5-His-TOPO vector (Invitrogen). MKN28 and Rat2 cells (2 × 10^5^ cells each) were transfected with 2 *μ*g of either RAMP-expressing plasmid or empty plasmid as control using Lipofectamine 2000 Transfection Reagent (Invitrogen). After incubation for 48 h, 500 *μ*g ml^−1^ G418 were added for selection. After 2 weeks, G418 resistant colonies were selected as stable transfectant cell lines.

### Cell proliferation assay

Cell proliferation was measured by the 3-(4,5-dimethylthiazol-2-yl)-5-(3-carboxymethoxyphenyl)-2-(4-sulphophenyl)-2H-tetrazolium (MTS) assay according to manufacture's instruction (Promega). Briefly, 20 *μ*l of reaction solution containing 333 *μ*g ml^−1^ MTS and 25 *μ*M phenazine ethosulphate was added to cells in 100 *μ*l culture medium. The mixture was incubated at 37°C for 1.5 h. The optical density (OD) was measured at a wavelength of 490 nm.

### Colony formation assay

Cells (1.0 × 10^4^) were mixed with 2 ml of culture medium containing 0.3% agar and 10% FBS and then plated on the bottom layer containing medium, and 0.6% agar with 10% FBS in each well of a six-well plate. After cultured for 21 days, colonies were counted after staining with crystal violet.

### Cell apoptosis assay

Cells were washed and resuspended in 100 *μ*l of annexin-binding buffer (1 × 10^6^ cells per ml), incubated with 5 *μ*l of annexin V conjugate (Invitrogen) and 100 *μ*g ml^−1^ of propidium iodide (PI) for 15 min at room temperature. After adding 400 *μ*l of annexin-binding buffer, flow cytometry was performed by FACScan (Becton Dickinson, Franklin Lakes, NJ, USA). The data were analysed by WinMDI 2.8 software (http://facs.scripps.edu/software.html).

### Cell-cycle analysis

The harvested cells were fixed in 1 ml of 70% cold ethanol at 4°C overnight, incubated with 10 *μ*g ml^−1^ RNase and stained with 50 *μ*g ml^−1^ PI for 30 min in the dark at 37°C. Samples were analysed by FACScan. The cell-cycle distribution was analysed by Modfit LT sofware.

### Western blot analysis

Cell pellets were lysed with Cyto Buster Protein Extraction Reagent (EMD Biosciences, San Diego, CA, USA). Protein concentration was measured with the Bio-Rad Protein Assay kit (Bio-Rad, Hercules, CA, USA). Forty *μ*g of eachProtein samples (40 *μ*g each) were separated by SDS–PAGE and transferred onto a nitrocellulose membrane (Amersham Biosciences, Piscataway, NJ, USA). Membranes were probed with specific antibodies including anti-RAMP (1 : 500), anti-p53 (1 : 1000), anti-p21 (1 : 1000), anti-cleaved caspase-3 (1 : 1000), anti-cleaved PARP (1 : 1000), anti-caspase-8 (1 : 1000), anti-caspase-9 (1 : 1000), anti *β*-actin (1 : 5000; Cell Signaling) and anti-GAPDH (1 : 10 000; Santa Cruz Biotechnology, Santa Cruz, CA, USA), respectively, and then incubated with secondary antibodies conjugated with horseradish peroxidase after washing. The signals were visualised with Super Signal ECL (Pierce Biotechnology, Rockford, IL, USA).

### Statistical analysis

Statistical analysis was performed using Student's *t*-test, *χ*^2^-test or Wilcoxon signed-rank test where appropriate. All *P* values are two sided and a value of less than 0.05 was considered statistically significant. All statistical calculations were performed by the SPSS software (version 13.0, Chicago, IL, USA).

## Results

### RAMP was upregulated in human gastric cancer cell lines and primary gastric cancer tissues

**Figure 1:**
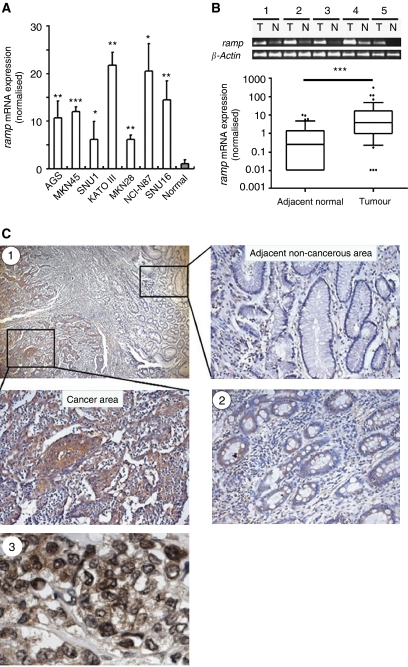
mRNA expression levels of *ramp* in (**A**) human gastric cancer cell lines and (**B**) primary gastric cancer and their adjacent non-cancerous tissues were determined by quantitative real-time PCR. The results were expressed as the ratio of copies of *ramp* relevant to *β-actin* from at least three independent experiments. Data are expressed as mean±s.d.; ^*^P<0.05, ^**^P<0.01, ^***^P<0.0001. (**C**) Representative immunohistochemical staining of RAMP protein expression in gastric cancer (**C1**) and intestinal metaplasia (**C2**). (**C3**) Subcellular localisation of RAMP protein in human cancer cells. Black arrowheads indicate some examples of cancer cells with RAMP expression.

To investigate whether RAMP might be involved in gastric carcinogenesis, the mRNA expression of *ramp* was first examined in 7 human gastric cancer cell lines (MKN45, AGS, SNU1, MKN28, NCI-N87, SNU16 and KATO III) and 47 gastric cancer tissues. *Ramp* mRNA was highly expressed in all seven cell lines (*P*<0.05; Figure 1A). Of the 47 paired gastric tumour tissues analysed, 38 cases (81%) showed increased *ramp* mRNA expression compared with their adjacent non-cancerous tissues (*P*<0.001, Wilcoxon test; Figure 1B). Consistent with the mRNA expression, strong and dense RAMP immunoreactivity was presented in gastric tumour tissues, whereas RAMP immunoactivity was only noted occasionally in adjacent non-cancerous tissues (Figure 1C1). Ramp protein expression was identified in both nuclear and cytoplasmic regions of gastric cancer cells (Figure 1C3). Furthermore, moderate level of RAMP expression was detected in precancerous gastric lesion, intestinal metaplasia (Figure 1C2).

### Association between RAMP expression and clinicopathological characteristics of gastric cancers

Among the 150 gastric cancer tissues evaluated on tissue array analysis, we found that RAMP protein expression was significantly higher in intestinal type gastric cancer than in diffuse type gastric cancer (*P*<0.01, *χ*^2^-test; Supplementary Table 1). However, there was no correlation between RAMP protein expression and other clinicopathological features such as gender, *H. pylori* infection and tumour stage.

### Knockdown RAMP expression inhibited tumour cell growth

**Figure 2:**
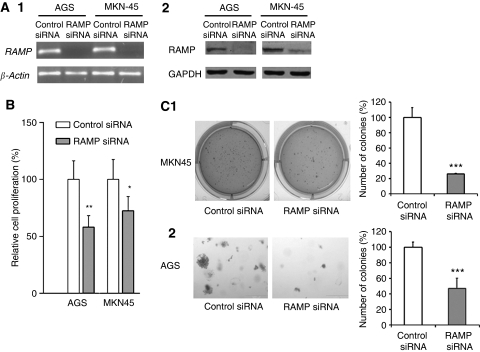
Knockdown expression of RAMP in AGS and MKN45 cell lines was confirmed by (**A1**) RT–PCR and (**A2**) western blotting. (**B**) Knockdown RAMP significantly inhibited tumour cell proliferation. (**C**) Knockdown RAMP significantly suppressed cell growth as determined by colony formation assay. Values are expressed as the mean±s.d. from three independent experiments; ^*^P<0.05, ^**^P<0.01, ^***^P<0.0001.

Having shown that RAMP was overexpressed in gastric cancer, we further investigated whether RAMP is casually involved in tumour cell growth. As shown in Figure 2, knockdown RAMP expression (Figure 2A) caused a significant decrease in cell proliferation rate in AGS (41.7%, *P*<0.01, *t*-test) and MKN-45 cells (27.2%, *P*<0.05, *t*-test; Figure 2B). This effect was further confirmed by colony formation assays. RAMP knockdown significantly reduced the colony formation efficiencies in MKN45 cells (*P*<0.001, *t*-test; Figure 2C1) and AGS cells (*P*<0.001, *t*-test; Figure 2C2) relative to corresponding controls, indicating that RAMP knockdown suppressed tumour cell growth.

### Knockdown RAMP expression caused cell cycle-arrest in G_2_/M phase

**Figure 3:**
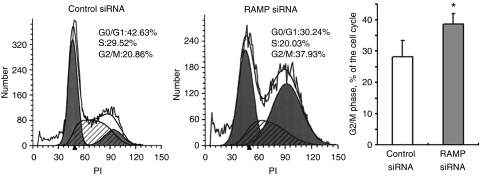
Effect of knockdown RAMP on cell-cycle distribution. Knockdown RAMP increased the number of G_2_/M phase cells by flow cytometry. Values are expressed as the mean±s.d. of three replicate experiments; ^*^P<0.05.

Concomitant with the suppression of cell proliferation, RAMP knockdown in AGS cells caused cell-cycle arrest in G_2_/M phase from 28.18±4.14% to 38.58±3.35% (*P*<0.05; Figure 3).

### Knockdown RAMP expression induced cell apoptosis

**Figure 4:**
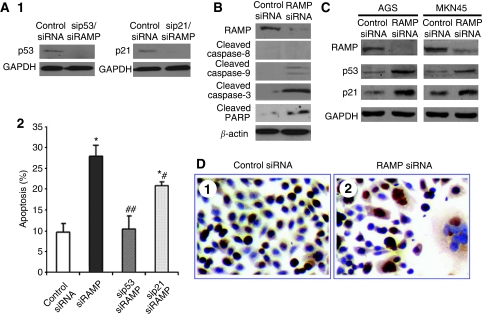
Effect of RAMP knockdown on apoptosis. (**A**) Double knockdown of RAMP/p53 and RAMP/p21 was performed in AGS cell. (**A1**) P53 and p21 knockdown was confirmed by western blot. (**A2**) Effect of knockdown RAMP, double knockdown RAMP/p53 or double knockdown RAMP/p21 on apoptosis of AGS cells, as determined by annexin V staining followed by flow cytometry. Values are expressed as the mean±s.d. of three replicate experiments. ^*^P<0.01 compared with control siRNA; #*P*<0.01 compared with siRAMP. (**B**) Western blotting of cleaved caspase-8, caspase-9 and caspase-3 and cleaved PARP in AGS cells transfected with RAMP siRNA. (**C**) Protein expression of p53 and p21 in AGS and MKN45 cells on reduced RAMP expression. (**D**) Representative images of immunocytochemical staining of p53 in AGS cells after RAMP siRNA transfection.

To investigate whether the decreased cell growth after RAMP knockdown was associated with induction of apoptosis, cell apoptosis was evaluated by annexin V staining followed by flow cytometry. RAMP knockdown induced apoptosis in AGS cells from 9.61±2.11 to 27.83%±2.69 (*P*<0.001; Figure 4A2). Induction of apoptosis was further confirmed by western blot analysis on caspase-3, a key factor in apoptosis execution, its downstream target PARP and two upstream modulators caspase-8 and caspase-9. As shown in Figure 4B, both caspase-3 and PARP were cleaved in RAMP knockdown AGS cells. Cleaved caspase-9 was also observed in AGS cells after RAMP expression was reduced, suggesting that mitochondrial death pathway was activated to mediate the cell death (Figure 4B). However, attenuated RAMP expression did not trigger cleavage of caspase-8, suggesting that the observed cell death may not be induced by the death-receptor pathway (Figure 4B). Moreover, RAMP knockdown resulted in increase of proapoptotic proteins, p53 and p21, in both AGS and MKN45 cells (Figure 4C). Immunocytochemical staining of p53 revealed that upregulation of p53 located in nuclear only region (Figure 4D), indicating the functional upregulation of p53 was achieved by RAMP knockdown. In addition, multi-nuclear giant tumour cells were also observed in RAMP knockdown AGS cells (Figure 4D2). This finding is consistent with a previous study suggesting that one possible function of RAMP is to mediate cytokinesis (Ueki *et al*, 2008). When we depleted RAMP expression with siRNA in AGS cells, it caused accumulation of G_2_/M cells. Taken together, our observation of multi-nuclear giant tumour cells could be due to deficit in cytokinesis. We further investigated the role of RAMP on cell apoptosis in relation to p53 and p21, a double knockdown of RAMP/p53, or RAMP/p21 was performed on AGS cells (Figure 4A). We found that apoptosis induced by RAMP knockdown was completely abolished by RAMP/p53 knockdown, but partially abolished by RAMP/p21 knockdown (Figure 4A), suggested that RAMP-mediated cell apoptosis is in p53- and p21-dependent pathways.

### Forced RAMP expression in MKN28 and Rat2 cells increased the clonogenicity in soft agar

**Figure 5:**
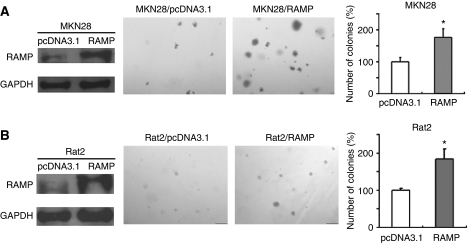
Overexpression of RAMP promoted cell colony formation in soft agar assay. (**A**) MKN28 and (**B**) Rat2 were stably transfected with RAMP-expressing or empty vectors. Expression of RAMP in transfected cells was confirmed by western blotting. Assays were performed in triplicate for three times. Quantitative analyses of colony numbers are shown as values of mean±s.d.; ^*^P<0.05.

To further investigate the oncogenic potential of RAMP in gastric cancer, we examined the effect of RAMP overexpression on growth characteristics of gastric tumour cells using soft agar colony formation assays. RAMP expression vector was stably transfected into MKN28 cells, which exhibits the lowest RAMP expression among seven gastric cancer cell lines, and Rat2 cells, an immortalised normal rat fibroblast cell line. Overexpression of RAMP in the transfected cells was confirmed by western blotting (Figure 5). Our results demonstrated that the colony formation efficiencies from the RAMP-transfected MKN28 and Rat2 cells were significantly higher (*P*<0.05) and larger in size than those of empty vector transfected cells (Figure 5).

## Discussion

This is the first study to establish a possible link between RAMP and gastric carcinogenesis. In this study, we found a significant increase in the expression of RAMP mRNA and protein in human gastric cancers compared to surrounding non-cancerous tissues. We also found that RAMP protein was moderately expressed in intestinal metaplasia (gastric precancerous lesion). Interestingly, higher expression of RAMP protein was detected in intestinal-type gastric adenocarcinoma compared to the diffuse type in our tissue array data. Intestinal-type gastric cancer usually happens at a late age and arises from chronic gastritis and develops through intermediate stages of atrophic gastritis, intestinal metaplasia, dysplasia and finally gastric cancer (Peek and Blaser, 2002). Diffuse-type gastric adenocarcinoma, on the other hand, consists of individually infiltrating neoplastic cells that do not form glandular structures and are not associated with intestinal metaplasia. Our findings suggest that frequent upregulation of RAMP may play a pivotal role in multi-step development of gastric cancer, and RAMP upregulation occurs as early as intestinal metaplasia throughout the gastric carcinogenesis.

**Figure 6:**
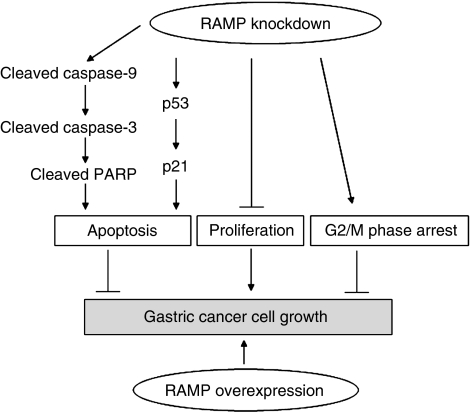
Schematic diagram for the mechanisms of RAMP function in gastric cancer cells. Knockdown of RAMP inhibited gastric cancer cells growth, which was associated with several biological effects: (1) increasing the expression of cleaved caspase-9, caspase-3 and PARP, which in turn induced apoptosis; (2) inducing apoptosis caused by knockdown of RAMP was dependent on p53 and p21 pathways; (3) suppressing cell proliferation; (4) causing cell arrest in G_2_/M phase. On the other hand, overexpression of RAMP promoted growth capacity. Thus, RAMP may function as a novel oncogene in gastric cancer.

Upregulation of RAMP in gastric cancer cells and primary gastric cancer tissues prompted us to elucidate the role of RAMP in gastric cancer. We therefore analysed the effects of knocking down RAMP expression in two human gastric cancer cell lines. Knockdown of RAMP by siRNAs inhibited cell proliferation and anchorage-independent growth in soft agar in gastric cancer cells (Figure 6). In accordance with our findings, other reports indicated that RAMP plays a crucial role in cell survival, and complete loss of RAMP is lethal in early mouse embryonic development (Liu *et al*, 2007).

We further characterised the functional significance of RAMP in cell-cycle changes and apoptosis. Our data indicated that knockdown of RAMP markedly induced cell apoptosis and caused cell-cycle arrest in G_2_/M phase. These changes are associated with induction of caspase-9 and caspase-3 cleavage, PARP cleavage and upregulation of p53 and its downstream target p21 (Wang and Prives, 1995; Chavez-Reyes *et al*, 2003). As it was suggested that RAMP and PCNA contribute to substrate recognition of p53 in the DDB1/CUL4 ligase complex (Banks *et al*, 2006; Higa *et al*, 2006a, 2006b), inhibition of RAMP largely impaired the function of DDB1/CUL4 ligase which profoundly stabilised p53 by preventing from ubiquitination and increased the level of p53 and its target p21. P53 regulates apoptosis and the cell cycle through actions in the nucleus. Altering the subcellular localisation of p53 can alter its biological functions (Moll *et al*, 2005). Our results showed that p53 was mainly retained in the nucleus after RAMP knockdown, indicating that p53 functioned as a transcription factor to increase the expression of p21 (McKenzie *et al*, 1999; Jiang *et al*, 2001; Jing *et al*, 2007) and promoted apoptosis (Yonish-Rouach *et al*, 1991; Lowe *et al*, 1993; Tsao *et al*, 1999). Moreover, the apoptosis induced by RAMP knockdown can be completely counteracted by RAMP/p53 double knockdown, but only partially abolished by RAMP/p21 double knockdown. These observations suggest that RAMP-mediated apoptosis in gastric cancer cells is dependent on p53 pathway or at least partly dependent on p21 pathway. In this regard, knockdown of RAMP increases functional p53 and p21 protein expression and induced cleavage of caspase-9, caspase-3 and PARP, which contributes to promote apoptosis (Nicholson *et al*, 1995; Oliver *et al*, 1998). We further classified all seven gastric cancer cell lines based on the p53 mutation status: MKN45 (p53-wildtype), AGS (p53-wildtype), SNU1 (p53-wildtype), MKN28 (p53-mutant), NCI-N87 (p53-mutant), SNU16 (p53-mutant) and KATO III (p53-deficient; Matozaki *et al*, 1992; Yokozaki, 2000; Jiang *et al*, 2001). However, we could not observe obvious correlation between RAMP expression levels and p53 mutation status (Figure 1A). Together with the fact that it has been suggested that RAMP regulates p53 expression, these observations further suggest that aberrant upregulation of RAMP in gastric cancer is an upstream event of the p53-dependent signalling.

To further characterise the functional significance of RAMP in gastric cancer, we also examined the effect of RAMP overexpression in gastric cancer cells. Strikingly, our results demonstrated that stable transfection of exogenous RAMP protein in both MKN28 and Rat2 cells promoted malignant transformation phenotypes as featured by the anchorage-independent growth of cancer cells in soft agar. These results provide additional evidence that RAMP played a pivotal role in promoting gastric cancer growth.

In conclusion, human gastric cancers exhibited higher RAMP expression in relation to surrounding tissue. Knockdown of RAMP inhibited cell proliferation, induced apoptosis and arrested cells in G_2_/M phase. We also provided further evidence that RAMP knockdown induced expression of cleaved caspase-9 and cleaved caspase-3, cleaved PARP and functional p53 and p21 protein in gastric cancer cells. On the other hand, overexpression of RAMP increased growth capacity in gastric cancer cells (Figure 6). Collectively, RAMP could play an important role in gastric carcinogenesis with an oncogenic potential. Inhibition of RAMP may be a novel approach for gastric cancer therapy that warrants future investigation.

## References

[bib1] Angers S, Li T, Yi X, MacCoss MJ, Moon RT, Zheng N (2006) Molecular architecture and assembly of the DDB1-CUL4A ubiquitin ligase machinery. Nature 443: 590–593 16964240 10.1038/nature05175

[bib2] Banks D, Wu M, Higa LA, Gavrilova N, Quan J, Ye T, Kobayashi R, Sun H, Zhang H (2006) L2DTL/CDT2 and PCNA interact with p53 and regulate p53 polyubiquitination and protein stability through MDM2 and CUL4A/DDB1 complexes. Cell Cycle 5: 1719–1729 16861890 10.4161/cc.5.15.3150

[bib3] Callagy G, Cattaneo E, Daigo Y, Happerfield L, Bobrow LG, Pharoah PD, Caldas C (2003) Molecular classification of breast carcinomas using tissue microarrays. Diagn Mol Pathol 12: 27–34 12605033 10.1097/00019606-200303000-00004

[bib4] Chavez-Reyes A, Parant JM, Amelse LL, de Oca Luna RM, Korsmeyer SJ, Lozano G (2003) Switching mechanisms of cell death in mdm2- and mdm4-null mice by deletion of p53 downstream targets. Cancer Res 63: 8664–8669 14695178

[bib5] Cheung WM, Chu AH, Chu PW, Ip NY (2001) Cloning and expression of a novel nuclear matrix-associated protein that is regulated during the retinoic acid-induced neuronal differentiation. J Biol Chem 276: 17083–17091 11278750 10.1074/jbc.M010802200

[bib6] Higa LA, Banks D, Wu M, Kobayashi R, Sun H, Zhang H (2006a) L2DTL/CDT2 interacts with the CUL4/DDB1 complex and PCNA and regulates CDT1 proteolysis in response to DNA damage. Cell Cycle 5: 1675–1680 16861906 10.4161/cc.5.15.3149

[bib7] Higa LA, Wu M, Ye T, Kobayashi R, Sun H, Zhang H (2006b) CUL4-DDB1 ubiquitin ligase interacts with multiple WD40-repeat proteins and regulates histone methylation. Nature Cell Biol 8: 1277–1283 17041588 10.1038/ncb1490

[bib8] Jiang XH, Wong BC, Lin MC, Zhu GH, Kung HF, Jiang SH, Yang D, Lam SK (2001) Functional p53 is required for triptolide-induced apoptosis and AP-1 and nuclear factor-kB activation in gastric cancer cells. Oncogene 20: 8009–8018 11753684 10.1038/sj.onc.1204981

[bib9] Jing Y, Wang M, Tang W, Qi T, Gu C, Hao S, Zeng X (2007) c-Abl tyrosine kinase activates p21 transcription via interaction with p53. J Biol chem 141: 621–626 10.1093/jb/mvm068 17339230

[bib10] Kurzik-Dumke U, Nrubsuer M, Debes A (1996) Identification of a novel Drosophila melanogaster heat-shock gene lethal (2) denticleless [l(2)dtl], coding for an 83-kDa protein. Gene 171: 163–170 8666267 10.1016/0378-1119(95)00885-3

[bib11] Liu CL, Yu IS, Pan HW, Lin SW, Hsu HC (2007) L2dtl is essential for cell survival and nuclear division in early mouse embryonic development. J Biol Chem 282: 1109–1118 17107960 10.1074/jbc.M606535200

[bib12] Lowe SW, Schmitt EM, Smith SW, Osborne BA, Jacks T (1993) p53 is required for radiation induced apoptosis in mouse thymocytes. Nature 362: 847–849 8479522 10.1038/362847a0

[bib13] Matozaki T, Sakamoto C, Matsuda K, Suzuki T, Konda Y, Nakano O, Wada K, Uchida T, Nishisaki H, Nagao M et al (1992) Missense mutations and a deletion of the p53 gene in human gastric cancer. Biochem Biophys Res Commun 182: 215–223 1370612 10.1016/s0006-291x(05)80133-0

[bib14] McKenzie PP, Guichard SM, Middlemas DS, Ashmun RA, Danks MK, Harris LC (1999) Wild-type p53 can induce p21 and apoptosis in neuroblastoma cells but the DNA damage-induced G_1_ checkpoint function. Clin Cancer Res 5: 4199–4207 10632361

[bib15] Moll UM, Wolff S, Speidel D, Deppert W (2005) Transcription-independent pro-apoptotic functions of p53. Curr Opin Cell Biol 17: 631–636 16226451 10.1016/j.ceb.2005.09.007

[bib16] Nicholson DW, Ali A, Thornberry NA, Vaillancourt JP, Ding CK, Gallant M, Gareau Y, Griffin PR, Labelle M, Lazebnik YA, Munday NA, Raju SM, Smulson ME, Yamin TT, Yu V, Miller D (1995) Identification and inhibition of the ICE/CED-3 protease necessary for mammalian apoptosis. Nature 397: 37–43 10.1038/376037a0 7596430

[bib17] Oliver FJ, de la Rubia G, Rolli V, Ruiz-Ruiz MV, de Murcia G, Marcia JM (1998) Importance of poly(ADP-ribose) polymerase and its cleavage in apoptosis. Lesson from an uncleavable mutant. J Biol Chem 273: 33533–33539 9837934 10.1074/jbc.273.50.33533

[bib18] Pan HW, Chou HY, Liu SH, Peng SY, Liu CL, Hsu HC (2006) Role of L2DTL, cell cycle-regulated nuclear and centrosome protein, in aggressive hepatocellular carcinoma. Cell Cycle 5: 2676–2687 17106265 10.4161/cc.5.22.3500

[bib19] Parkin DM, Pisani P, Ferlay J (1999) Estimates of the worldwide incidence of 25 major cancers in 1990. Int J Cancer 80: 827–841 10074914 10.1002/(sici)1097-0215(19990315)80:6<827::aid-ijc6>3.0.co;2-p

[bib20] Peek RM, Blaser MJ (2002) *Helicobacter pylori* and gastrointestinal tract adenocarcinomas. Nat Rev Cancer 2: 28–37 11902583 10.1038/nrc703

[bib21] Pfleger CM, Kirschner MW (2000) The KEN box: an APC recognition signal distinct from the D box targeted by Cdh1. Genes Dev 14: 655–665 10733526 PMC316466

[bib22] Rimm DL, Camp RL, Charette LA, Olsen DA, Provost E (2001) Amplification of tissue by construction of tissue microarrays. Exp Mol Pathol 70: 255–264 11418004 10.1006/exmp.2001.2363

[bib23] Tsao YP, Huang SJ, Chang JL, Hsieh JT, Pong RC, Chen SL (1999) Adenovirus-mediated p21^(WAF1/SDII/CIP1)^ gene transfer induces apoptosis of human cervical cancer cell lines. J Virol 73: 4983–4990 10233960 10.1128/jvi.73.6.4983-4990.1999 PMC112542

[bib24] Ueki T, Nishidate T, Park JH, Lin ML, Shimo A, Hirata K, Nakamura Y, Katagiri T (2008) Involvement of elevated expression of multiple cell-cycle regulator, DTL/RAMP (denticleless/RA-regulated nuclear matrix associated protein), in the growth of breast cancer cells. Oncogene 27: 5672–5683 18542055 10.1038/onc.2008.186

[bib25] Wang Y, Prives C (1995) Increased and altered DNA binding of human p53 by S and G2/M but not G1 cyclin-dependent kinases. Nature 376: 88–91 7596441 10.1038/376088a0

[bib26] Yokozaki H (2000) Molecular characteristics of eight gastric cancer cell lines established in Japan. Pathol Int 50: 767–777 11107048 10.1046/j.1440-1827.2000.01117.x

[bib27] Yonish-Rouach E, Resnitzky D, Lotem J, Sachs L, Kimchi A, Oren M (1991) Wild-type p53 induces apoptosis of myeloid leukaemic cells that is inhibited by interleukin-6. Nature 352: 345–347 1852210 10.1038/352345a0

