# Supplementary material for: Identification of retinoic acid-regulated nuclear matrix-associated protein as a novel regulator of gastric cancer
Source: Br J Cancer. 2009 Aug 11;101(4):691–8. doi: 10.1038/sj.bjc.6605202 (PMC2736823; doi:10.1038/sj.bjc.6605202)
Supplement: Supplementary Table 1 [file 6605202x1.doc]

**Table 1 Clinicopathologic features of RAMP expression in gastric cancer (*n* = 150)**

| **Characteristics** | **Low score of expression*** | **High score of expression*** | ***p-value (χ^2^)*** |
| --- | --- | --- | --- |
|  | ***n*** | ***n*** |  |
| **Age** |  |  |  |
| Mean ± SD | 64.1±12.9 | 70.4± 10.5 |  |
| **Gender** |  |  |  |
| M | 66 | 29 | 0.551 |
| F | 38 | 17 |
| **H. pylori infection** |  |  |  |
| Positive | 31 | 17 | 0.315 |
| Negative | 59 | 25 |
| **Lauren** |  |  |  |
| Diffuse | 46 | 9 | 0.005 |
| Intestinal | 52 | 34 |
| **Differentiation** |  |  |  |
| Poor (or no differentiation) | 52 | 20 | 0.541 |
| Moderate | 38 | 22 |
| Well | 5 | 2 |
| **TNM stage** |  |  |  |
| I | 11 | 8 | 0.728 |
| II | 15 | 6 |
| III | 40 | 17 |
| IV | 37 | 15 |

* A histochemical score was obtained by multiplying the intensity of staining by the percentage of positive cancer cells. For the intensity, a score of 1 to 3, corresponding to weak, moderate and strong positivity, was recorded. The percentage of positive cells at each intensity was also estimated. The score is calculated as 1 x weak % + 2 x moderate % + 3 x strongly stained %. The range of possible scores of 0 to 3 represents low score of expression and 4 to 9 represents high score of expression.
